# Surface-Dependent Isotopic Adsorption of CO on α-Al_2_O_3_: Role of Weak Interactions and Zero-Point Energy

**DOI:** 10.3390/molecules30092067

**Published:** 2025-05-06

**Authors:** Qun Yang, Xiyue Cheng, Qian Xu, Shuiquan Deng

**Affiliations:** 1 Key Laboratory of Optoelectronic Materials Chemistry and Physics, Fujian Institute of Research on the Stricture of Matter, Chinese Academy of Sciences, Fuzhou 350002, China; 2 College of Chemistry and Materials Science, Fujian Normal University, Fuzhou 350007, China; 3 Fujian College, University of Chinese Academy of Sciences, Beijing 100049, China; 4 State Key Laboratory of Structural Chemistry, Fujian Institute of Research on the Structure of Matter, Chinese Academy of Sciences, Fuzhou 350002, China

**Keywords:** isotope separation, surface adsorption, first-principles calculations

## Abstract

Carbon isotopes, particularly ^13^C, are critical for applications in food authentication, biomedical diagnostics, and metabolic research; however, their efficient separation remains challenging due to their low natural abundance. This study investigates the adsorption behavior of ^12^CO and ^13^CO on various low-index α-Al_2_O_3_ surfaces as a strategy for isotope separation. Density functional theory (DFT) calculations with D3 (BJ) dispersion corrections were employed to optimize surface models for five representative α-Al_2_O_3_ facets. Nine adsorption configurations were systematically evaluated by optimizing geometric structures, computing adsorption enthalpies with zero-point energy corrections, and performing Bader charge and charge density difference analyses to elucidate interfacial interactions. The results reveal that CO preferentially adsorbs in a vertical configuration via its carbon end at Al sites, with the (0001) surface exhibiting the lowest surface energy and most favorable adsorption characteristics. Furthermore, we found that facets with lower surface energy not only facilitate stronger CO adsorption but also demonstrate pronounced adsorption enthalpy differences between ^12^CO and ^13^CO, driven by vibrational zero-point energy disparities. These findings highlight the potential of low adsorption enthalpy surfaces, particularly (0001), ($01\bar{1}2$), and ($11\bar{2}0$), for enhancing isotope separation efficiency, providing valuable insights for the design of advanced separation materials.

## 1. Introduction

Carbon is a fundamental element in natural organic matter, existing primarily as two stable isotopes, ^12^C and ^13^C, with ^14^C being a radioactive isotope. Among them, ^13^C has attracted significant interest due to its non-radioactivity, chemical stability, and biological safety, facilitating its widespread application in diverse fields ranging from food authentication and biomedical diagnostics to metabolic research [1,2,3]. In clinical settings, ^13^C-labeled compounds serve as tracers in nuclear magnetic resonance (NMR) spectroscopy for real-time monitoring of metabolic pathways [4]. A prominent example is the ^13^C–urea breath test, a non-invasive diagnostic tool for detecting Helicobacter pylori infections, which relies on analyzing isotopic enrichment in exhaled CO_2_ [5]. In the agri-food sector, distinct *δ* ^13^C signatures between C_4_ plants and C_3_ plants are leveraged for origin verification and fraud detection in supply chains [6]. Despite its growing demand, the commercial-scale separation of ^13^C remains challenging due to its low natural abundance at 1.1%. Traditional isotope enrichment methods, such as cryogenic distillation, are energy-intensive, capital-demanding, and limited in throughput. These drawbacks underscore the critical need for more efficient, cost-effective, and scalable separation techniques to address the increasing industrial and research demands for high-purity ^13^C.

Recent advancements have highlighted the potential of porous materials in isotope separation, leveraging mechanisms like kinetic quantum sieving (KQS) [7] and chemical affinity quantum sieving (CAQS) [8]. Experimental investigations in the field of hydrogen/deuterium (H/D) isotope separation have confirmed the effectiveness of porous materials as selective adsorbents. The KQS effect occurs at cryogenic temperatures (20–50 K) when pore sizes approach the molecular de Broglie wavelength, leading to quantum energy barriers that favor diffusion of heavier isotopes such as D_2_ over H_2_ due to lower zero-point energy [7]. Conversely, the CAQS effect operates without ultra-low temperatures, relying on differences in zero-point energy and binding affinity, with heavier isotopes preferentially adsorbing at stronger sites [8]. For instance, through the KQS effect, researchers have obtained an optimal separation factor of 13.6 for the H/D isotopes at 40 K [9], significantly surpassing the factor of 1.5 achieved by cryogenic distillation at 24 K [10]. In 2014, Hu et al. [11] investigated proton conduction through graphene, hBN, and MoS_2_, uncovering the proton transmission barriers. They found that decorating graphene films with catalytic metal nanoparticles enhances proton transport performance, resulting in high proton selectivity. In 2017, Kim et al. [12] reported that incorporating imidazole molecules into the open metal site channels of MOF-74 allowed the synergistic effects of KQS and CAQS to maximize separation performance, achieving a separation factor as high as 26 for H_2_ and D_2_ at 77 K. In 2022, Su et al. [13] designed a porous coordination polymer with dynamically tunable gates, achieving highly efficient separation of water isotope isomers (H_2_O/HDO/D_2_O) at room temperature with a water separation factor as high as 210.

For heavier isotopes, in 2021, Ujjain et al. [14] demonstrated that nanoporous carbide-derived carbon (CDC) exhibits extremely high adsorption selectivity for ^18^O_2_, with selectivity increasing as temperature decreases and being significantly affected by pore geometry; their adsorption–separation device based on CDC achieved a S(^18^O_2_/^16^O_2_) of approximately 60 ± 15 at 112 K and, similarly, a S(^13^C/^12^C) of 56 ± 6 for ^13^CH_4_ separation. Theoretically, in 2018, Tian et al. [15] explored the potential of MOF materials for carbon isotope separation through adsorption or membrane separation. Using classical density functional theory (cDFT) methods, they calculated the gas diffusion rates and isotope selectivities of methane pairs with different ^12^CH_4_/^13^CH_4_ carbon isotopes, demonstrating that MOFs are capable of separating carbon isotopes. Subsequent studies by Wang et al. [16] validated these results by screening 12,478 MOF materials, identifing nanoporous membranes with high selectivity for ^12^CH_4_/^13^CH_4_ separation. These experimental and theoretical studies prove the potential of using porous materials for the separation of carbon isotopes.

Beyond conventional porous materials such as molecular sieves, carbon-based materials, and MOFs, alumina as a porous metal oxide also exhibits a porous structure and strong polar adsorption capability [17]. With its nanoscale particle size, high specific surface area, and internal pore structure conducive to adsorbing isotope molecules, porous Al_2_O_3_ emerges as a promising candidate for isotope separation applications, which can potentially overcome the limitations of traditional techniques [18]. The coexistence of Lewis acid (Al^3+^) and Lewis base (O^2−^) sites on its surface provides diverse adsorption site options for polar molecules, such as CO and NO_x_ [19]. Moreover, Al_2_O_3_ has demonstrated excellent selectivity and stability in the adsorption of inert gases, further underscoring its potential for gas separation technologies [20]. Its adsorption behavior can be precisely controlled through the modulation of surface exposure and atomic reconstruction, thereby substantially enhancing its gas adsorption and catalytic performance [21,22,23,24]. Recent computational studies have validated the potential of α-Al_2_O_3_ in gas separation applications. For instance, Kojabad et al. [25] developed α-Al_2_O_3_ composite membranes that exhibit excellent CO_2_ selectivity over N_2_ and CH_4_ under both dry and humid conditions, while maintaining high gas permeance. Consistently, metal-functionalized α-Al_2_O_3_ surfaces such as Ni-decorated (0001) facets demonstrate a preferential adsorption of CO_2_ and CO from complex gas mixtures containing CO_2_, CO, CH_4_, and H_2_, thereby enabling the selective purification of CH_4_ and H_2_ due to the weaker interactions of the latter gases with the surface [26]. Hass et al. [27] also demonstrated that α-Al_2_O_3_ (0001) readily dissociates water through surface-mediated pathways, even without defects, confirming its strong interaction with polar adsorbates under varying surface conditions. In terms of CO adsorption, extensive theoretical investigations have identified stable adsorption configurations on various α-Al_2_O_3_ single-crystal surfaces. Casarin et al. [28] reported that CO chemisorbs most stably atop Lewis acid (Al^3+^) sites in a perpendicular geometry on α-Al_2_O_3_ (0001), with a computed C-O stretching frequency ($\nu_{c-o}$) of 2158 cm^−1^, indicating a 44 cm^−1^ blue shift, in qualitative agreement with IR data on CO adsorbed on alumina powders. Additionally, Rohmann et al. [29] investigated CO adsorption on α-Al_2_O_3_ (0001) via DFT-GGA and infrared spectroscopy, demonstrating that the adsorption energy and C-O vibrational frequency vary with surface coverage due to adsorption-induced surface relaxation. Although α-Al_2_O_3_ low-index surfaces have been widely studied for gas adsorption, systematic investigations into isotope adsorption and separation remain unexplored. A fundamental understanding of the atomic- and electronic-scale mechanisms governing isotope separation on these surfaces is urgently needed.

In this study, we evaluate CO isotope separation on five low-index α-Al_2_O_3_ surfaces. A comprehensive investigation was conducted on the adsorption configurations, geometric parameters, and adsorption enthalpies of ^12^CO and ^13^CO molecules on the α-Al_2_O_3_ (0001) surface. A corrected adsorption enthalpy approach considering zero-point energy (ZPE) corrections was applied in the calculation of adsorption enthalpies. Among the nine configurations we examined, the most favorable involved vertical C-end adsorption atop an Al site. Bader charge analysis, charge density difference, and non-covalent interaction analyses revealed subtle yet noticeable charge transfer between CO and the surface. Comparative results across the five surfaces indicate that lower-energy facets—particularly (0001), ($01\bar{1}2$), and ($11\bar{2}0$)—not only exhibit stronger CO binding but also show more distinct differences between ^12^CO and ^13^CO adsorption enthalpies, underscoring their promise for surface-engineered isotope separation.

## 2. Results and Discussion

### 2.1. Surface Energy of Al_2_O_3_ Surfaces

**Figure 1:**
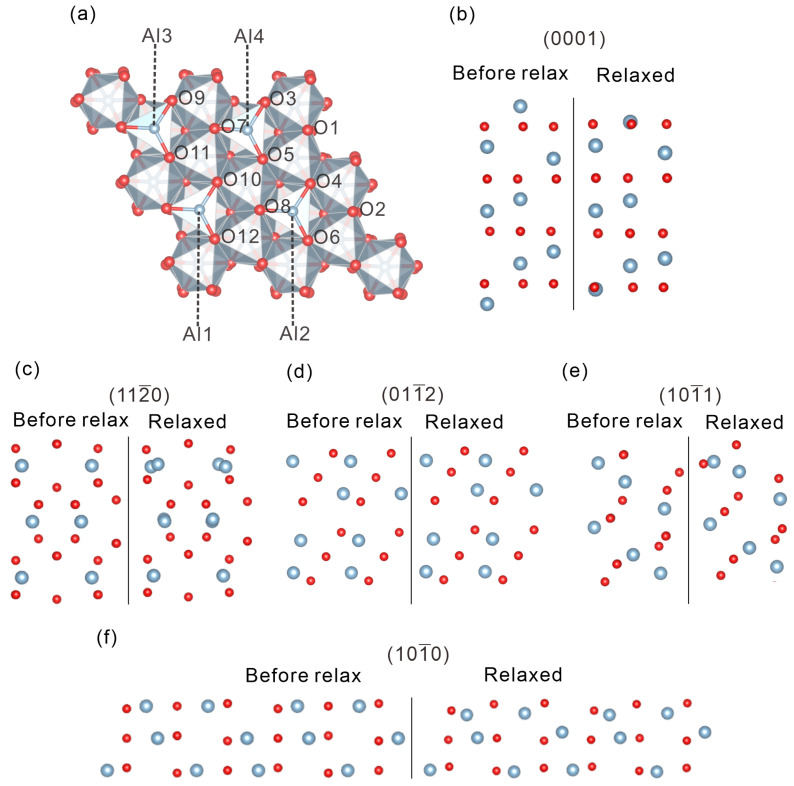
(**a**) Top view of the (0001) 2 × 2 surface showing 4 and 18 possible adsorption sites on Al and O atoms, respectively. (**b**–**f**) The positions of Al and O atoms in the unrelaxed and relaxed states for five low-index (0001), ($11\bar{2}0$), ($01\bar{1}2$), ($10\bar{1}1$), and ($10\bar{1}0$) surfaces, showing the positions of Al and O atoms in the repeating units of five low-index surfaces before and after relaxation. The diagrams illustrate the atomic configurations in the upper half of the layered structure, with the surface located at the top of each panel. Blue balls represent Al atoms, and red balls represent O atoms.

We primarily investigated the (0001), ($11\bar{2}0$), ($01\bar{1}2$), ($10\bar{1}1$), and ($10\bar{1}0$) surfaces of α-Al_2_O_3_ in this work. Although these surfaces can theoretically present a variety of configurations, our study assumes that all surfaces maintain stoichiometric balance and strictly adhere to the crystal symmetry, thereby precluding any surface reconstruction. Moreover, it is assumed that the unrelaxed surfaces are directly obtained by cleaving an ideal crystal along a single crystallographic plane [30]. In accordance with Tasker’s [31] rules, these surfaces are constructed with termination layers composed of repeating units that do not generate a net dipole moment. To construct a reliable model of an α-Al_2_O_3_ surface, we employed a layered geometric approach under periodic boundary conditions. Each supercell comprises multiple parallel slabs separated by a 20 Å vacuum layer along the c-axis. Each slab includes a sufficient number of repeating units—approximately 100 atoms per slab—to ensure convergence and computational stability. Surfaces models were generated from optimized bulk α-Al_2_O_3_ along the (0001), ($11\bar{2}0$), ($01\bar{1}2$), ($10\bar{1}1$), and ($10\bar{1}0$) planes. The specific stacking sequences were chosen based on the literature, as shown in Figure 1: For the (0001) surface, a repeating unit with the sequence M-3O-M··· (four layers) was employed [30,32,33]. We computed the surface energy $E_{surf}$ as a function of the number of Al-O-Al layers *n* in the slab (with n = 4–10) for (0001). The results are shown in Figure S1 of the Supplementary Materials. As one can see, $E_{surf}$ changes by less than 0.006 J/m^2^ upon increasing n from 4 to 10 and remains essentially constant for n ≥ 4. During the relaxation, we observe that the atomic displacement below the third layer is small, confirming that the displacements of the atoms in the deeper layers have a negligible effect on the overall result. Accordingly, we conclude that a four-layer (Al-O-Al) (0001) slab provides converged surface energies and captures all significant surface relaxations. For the ($11\bar{2}0$) surface, the sequence O-2O-4M-2O-O··· (7 layers) was adopted; for the ($01\bar{1}2$) surface, a 2O-2M-2O-2M-2O··· (7 layers) sequence was used; for the ($10\bar{1}1$) surface, the sequence O-M-O-M-O··· (10 layers) was employed; and for the ($10\bar{1}0$) surface, 2O-4M-2O-2O··· (8 layers) was constructed. These layer numbers were found to be sufficient to ensure convergence of the final results [34]. Significant differences in atomic displacements upon structural relaxation were observed among the five surfaces. As illustrated in Figure 1, the displacements of atomic layers parallel to the surface, with 3–4 layers of the repeating unit shown, reveal notable structural changes. For instance, on the (0001) surfaces, the Al atoms in the topmost repeating unit contract markedly inward, whereas on the ($10\bar{1}0$) surface, the Al atoms exhibit a pronounced inward movement, even penetrating the overlying O layer, demonstrating behavior consistent with that reported by Manassidis and Gillan [30]. In contrast, the atomic displacements on the other surfaces are considerably smaller.

The surface energies ($E_{surf}$) for these low-index surfaces was calculated using the following equation:

(1) $$E_{surf}={(E}_{\left(n\right)}-{nE}_{bulk})/(2A)$$

where A is the area of the surface, $E_{bulk}$ is the energy per unit layer of the bulk, and $E_{\left(n\right)}$ is the total energy of a slab containing n unit layers. The computed surface energies are summarized in Table 1. The results reveal the following order for the $E_{surf}$ of the relaxed surfaces, (0001) $<(11\bar{2}0)<(01\bar{1}2)<(10\bar{1}1)<$ ($10\bar{1}0$), which is in close agreement with the predictions reported by Sun [32]. Notably, the (0001) surface exhibits the lowest energy about 1.54 eV after relaxation, consistent with the findings of Sun et al. [32] and Mackrodt et al. [33] confirmed the validity of our computational methods. The α-Al_2_O_3_ (0001) surface exhibits the lowest energy among all facets investigated, thereby demonstrating its superior thermodynamic stability under equilibrium conditions. Consequently, the α-Al_2_O_3_ (0001) surface was selected for the subsequent adsorption site analysis.

### 2.2. Adsorption of ^12^CO and ^13^CO on α-Al_2_O_3_ ( 0001) Surface

**Figure 2:**
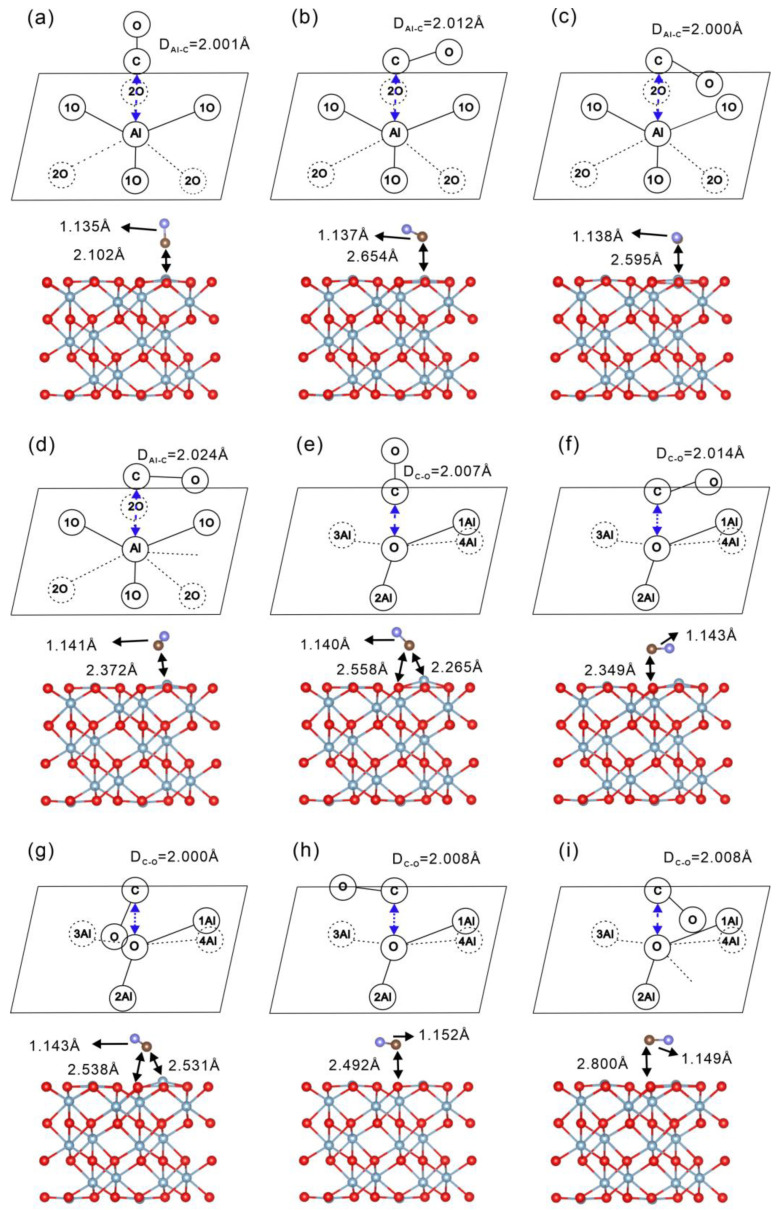
Nine adsorption configurations of CO adsorbed on the α-Al_2_O_3_ (0001) surface. Configurations (**a**–**d**) correspond to CO adsorption at the Al sites, while configurations (**e**–**i**) represent adsorption at the O sites. Configurations (**a**,**e**) feature a vertical adsorption geometry, whereas configurations (**b**–**d**) and (**f**–**i**) exhibit a horizontal adsorption orientation. Red and purple balls represent O atoms in α-Al_2_O_3_ and CO, respectively; blue balls represent Al atoms, and brown balls represent C atoms. The single-headed arrows indicate the C-O bond length, and the double-headed arrows indicate the distance between the C atom in CO and the surface Al or O atoms.

Geometric optimization of the gas-phase CO molecule by applying a 10 × 10 × 10 Å^3^ unit cell and a C-O bond length of 1.143 Å, approximately 0.015 Å longer than the experimental values of 1.128 Å [38]. This slight overestimation is attributable to the adopted exchange–correlation functional, basis-set incompleteness and finite plane-wave cutoff energy. To determine the most stable adsorption structure of CO on α-Al_2_O_3_, we systematically examined a series of adsorption configurations on the (0001) surface. Nine representative configurations were constructed at the top sites of the α-Al_2_O_3_ (0001) surface. Specifically, for adsorption on Al sites, calculations were centered on site 3 (as indicated in Figure 1a), while for adsorption on O sites, site 5 was selected. Figure 2 presents both schematic representations of the pre-adsorption configurations and side views of the optimized post-adsorption geometries for nine distinct configurations. Configurations (a–d) correspond to CO adsorption at Al sites, with configuration (a) adopting a vertical orientation and configurations (b–d) corresponding to horizontal adsorption. Similarly, configurations (e–i) represent CO adsorption at O sites, where configuration (e) is vertically adsorbed and configurations (f–i) exhibit horizontal geometries. In addition to visualizing the adsorption geometries, the figure also includes key structural parameters, including the initial distance between the carbon atom and surface Al or O atoms, as well as the corresponding bond lengths and adsorption distances after structural optimization. Adsorption via the oxygen end of CO on either Al or O sites was not considered in this study, as previous studies [28] have indicated that such configurations result in weak interactions with the surface and are therefore energetically unfavorable.

Based on static DFT-D3 (BJ) calculations, the interaction energies ($∆E_{int}$) were calculated by Equation (2):

(2) $$∆E_{int}=E_{{Al}_{2}O_{3}+CO}-E_{{Al}_{2}O_{3}}-E_{CO}$$

where $E_{{Al}_{2}O_{3}+CO}$ is the energy of the optimized α-Al_2_O_3_/CO system, $E_{{Al}_{2}O_{3}}$ refers to the energy of pristine α-Al_2_O_3_ structure, and $E_{CO}$ is the energy of a free CO molecule in the gas phase. The adsorption enthalpy ($∆H_{ads}$) at a given temperature *T* and standard pressure was then obtained by incorporating the zero-point energy ($∆E_{ZPE}$) correction, as shown in Equation (3):

(3) $$∆H_{ads}\left(T\right)=∆E_{int}+∆E_{ZPE}+∆E_{TE}(T)$$

In Equation (3), $∆E_{ZPE}$ represents the zero-point energy difference between the optimized α-Al_2_O_3_/CO system and a single gas-phase CO molecule, and $∆E_{TE}(T)$ is the thermal energy contribution. The temperature-dependent vibrational enthalpy was initially calculated to obtain $∆E_{TE}\left(T\right)$, but since its effect on the adsorption enthalpy was found to be minor at 0 K, it was not considered in the subsequent analysis. In the harmonic frequency analysis, the α-Al_2_O_3_ slab remained fixed at its optimized geometry and only the CO vibrational modes were considered. The zero-point energy is defined as follows [39]:

(4) $$E_{ZPE}=\frac{1}{2}\sum_{i=1}^{3N-3}hv_{i}$$

where h represents Planck’s constant, $v_{i}$ is the vibrational frequency of the *i*-th vibrational mode, and *N* denotes the number of atoms in the system. Under harmonic approximation, the vibrational frequencies are first computed, and $∆E_{ZPE}$ is subsequently evaluated as one-half of the summation of all the vibrational frequencies. In the present study, vibrational frequency calculations for the CO adsorption on alumina were carried out using VASP. The ZPE was then calculated from the vibrational frequencies according to Equation (4), with vibrational data extracted and processed using VASPKIT. [40]. The adsorption enthalpy $∆H_{ads}\left(T\right)$ and zero-point energy difference $∆E_{ZPE}$ for ^12^CO and ^13^CO at various sites are calculated as shown in Table 2. Lower adsorption enthalpy values indicate stronger adsorption capabilities. Notably, configurations (a) and (e) exhibit the most negative adsorption enthalpies at −0.6035 eV and −0.5754 eV, indicating these configurations possess the highest adsorption stability, with configuration (a)’s vertical adsorption on an Al site being the most favorable. Analysis of the schematic adsorption sites suggests that configurations (a) and (e) correspond to scenarios in which the CO molecule is adsorbed with its carbon atom oriented vertically toward an Al site. Furthermore, by comparing the relaxed Al-C distance, configuration (a) exhibits the shortest distance among the nine models, with a value of 2.102 Å. This suggests that the Al-C interactions in the other models are weaker than that in configuration (a). Therefore, this vertical configuration in the (a) model demonstrates the highest stability among all tested sites, as indicated by its shorter C-Al distance and lower adsorption enthalpy. In contrast, other parallel adsorption configurations also exhibit relatively high adsorption enthalpy values, indicating that adsorption in a parallel orientation is generally less stable. It is consistent with reports indicating that the C end of CO, when perpendicular to the adsorption site, is more readily adsorbed [41,42,43]. Furthermore, from configurations (e) and (g), it is observed that when the C atom of CO is initially positioned above a surface O atom, the molecule tends to reorient during structural optimization and is eventually attracted toward a nearby Al site. This further confirms the strong preference of CO to bind through the carbon end to Al atoms rather than O atoms, driven by stronger electrostatic and orbital interactions. Based on these findings, the subsequent studies on adsorption behavior across other α-Al_2_O_3_ surfaces will utilize models in which the carbon ends of both ^12^CO and ^13^CO are oriented perpendicular to the Al adsorption site.

### 2.3. Bader Charge and Electronic Structure Analysis

To understand the charge variations resulting from CO adsorption and the intricate interaction mechanism with the α-Al_2_O_3_ (0001) surface, we conducted the Bader charge analysis across all adsorption configurations. Table 3 summarizes the net charge transfer associated with CO adsorption, where a positive value indicates electron loss and a negative value indicates electron gain. Given that the chemical properties of ^12^CO and ^13^CO are nearly identical, the Bader charge and electronic structure analyses yield indistinguishable results for the Al_2_O_3_/^12^CO and Al_2_O_3_/^13^CO systems. Consequently, the discussion herein refers collectively to both isotopic systems. Among these nine configurations, the observed charge transfer values are as follows: 0.03e for configurations (a) and (d), 0.01e for configuration (b), 0.02e for configurations (c) and (i), 0.05e for configuration (e), 0.08e for configuration (f), and 0.04e for configuration (h). Overall, the electron transfer from the α-Al_2_O_3_ surface to the CO molecule is minimal, indicating only a subtle redistribution of charge.

**Figure 3:**
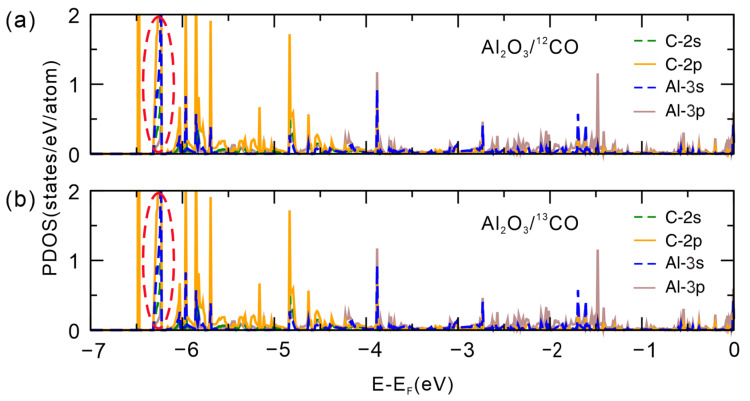
PDOS for the interaction between the C atom of CO and the Al atoms of the α-Al_2_O_3_ (0001) surface in (**a**) Al_2_O_3_/^12^CO and (**b**) Al_2_O_3_/^13^CO system. The red dashed circles indicate the region where hybridization occurs between the Al 3s and C 2p orbitals in the Al_2_O_3_/^12^CO and Al_2_O_3_/^13^CO systems.

To further elucidate the interaction mechanism between the α-Al_2_O_3_ (0001) surface and the CO molecules, we performed a partial density of states (PDOS) analysis in Figure 3. The PDOS plots at the top valence bands, focusing specifically on the C and Al atomic contributions, reveal a localized interaction predominantly within the −4.5 to −6.5 eV energy window. Notably, at approximately −6.3 eV, both the Al_2_O_3_/^12^CO and Al_2_O_3_/^13^CO systems exhibit pronounced hybridization, mainly between the Al 3s and C 2p orbitals. The observed orbital overlap, though limited, suggests that the interaction is not purely governed by van der Waals forces. Instead, a weak electronic hybridization between CO and the surface contributes to a slight bonding character, which is stronger than simple physisorption but much weaker than a typical covalent bond.

**Figure 4:**
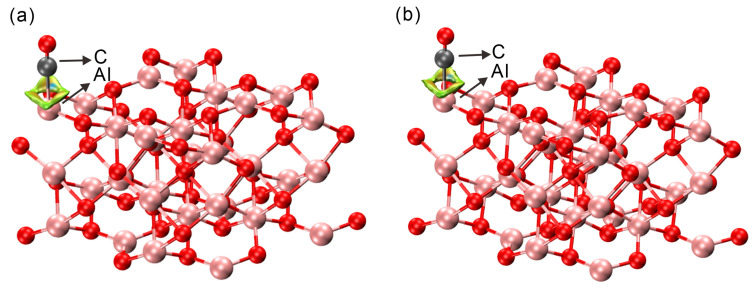
NCI plots of (**a**) ^12^CO and (**b**) ^13^CO molecules adsorbed on an α-Al_2_O_3_ (0001) surface. Pink balls represent Al atoms, red balls represent O atoms, and gray balls represent C atoms.

Furthermore, non-covalent interaction (NCI) analysis was conducted to gain deeper insight into the nature of the CO–surface interaction. NCI is a visualization technique based on the electron density and its gradient, which highlights regions of weak interactions—such as van der Waals forces—through low electron density and reduced density gradient values. In this study, NCI analysis was performed using the Critic2 program, version 1.2.2 [44], which supports both molecular and periodic systems. As shown in Figure 4, green to yellow isosurfaces are observed between the CO molecule and the α-Al_2_O_3_ (0001) surface. These features indicate the presence of weak attractive forces, mainly dispersion interactions. However, the subtle variation in isosurface color suggests that the interaction is not purely dispersive and may involve a small degree of charge redistribution. The absence of pronounced blue or red regions around the adsorption site indicates that there is neither strong electrostatic attraction nor significant steric repulsion. Combined with the minimal charge transfer and weak orbital hybridization observed from Bader analysis and PDOS, these results suggest that CO adsorption on the α-Al_2_O_3_ (0001) surface is primarily characterized by weak physisorption with minor electronic contributions beyond van der Waals forces.

### 2.4. Comparison of ^12^CO and ^13^CO Separation Performance on Different α-Al_2_O_3_ Surfaces

**Figure 5:**
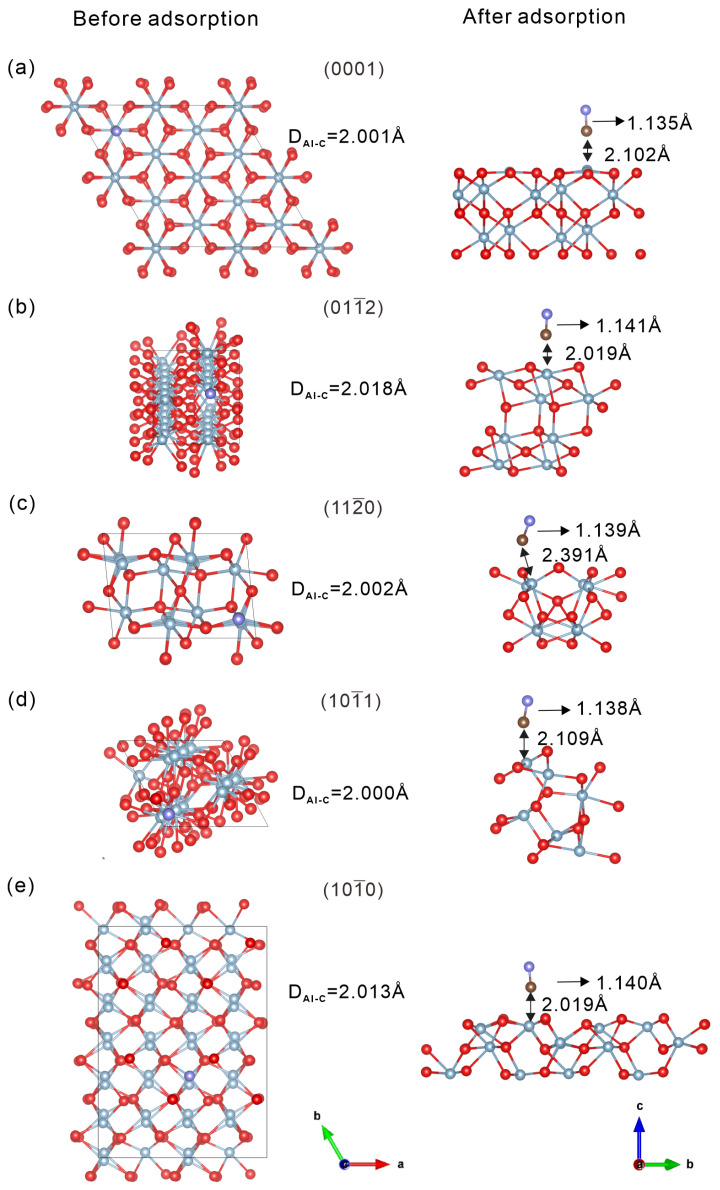
(**a**–**e**) Adsorption models of CO on five low-index α-Al_2_O_3_ (0001), ($01\bar{1}2$), ($11\bar{2}0$), ($10\bar{1}1$), and ($10\bar{1}0$) surfaces. For each surface, the left row shows the initial position of the CO molecule before geometry optimization (top view), while the right row shows the optimized structure after adsorption (side view). Red and purple balls represent O atoms in α-Al_2_O_3_ and CO, respectively; blue balls represent Al atoms, and brown balls represent C atoms. The single-headed arrows indicate the C-O bond length, and the double-headed arrows indicate the distance between the C atom in CO and the surface Al or O atoms.

The separation performance of different α-Al_2_O_3_ surfaces was evaluated through calculations of adsorption enthalpy, geometric parameters, and CO adsorption configurations at surface Al sites. As depicted in Figure 5, the top view represents the initial adsorption geometry of the CO molecule prior to interaction with the surface, whereas the side view illustrates the fully relaxed configuration after adsorption. Structural parameters such as the initial C–Al distance (D_Al-C_), the C–O bond length, and the final D_Al–C_ after relaxation are labeled to highlight adsorption-induced changes. These geometric descriptors provide insight into the adsorption mechanism and the extent of structural reorganization upon binding.

As shown in Table 4, the adsorption enthalpies of ^13^CO are consistently slightly lower than those of ^12^CO across all examined surfaces, with differences ranging from 1.51 to 1.85 meV. This consistent trend suggests that ^13^CO interacts slightly more strongly with the surface, indicating a small but measurable isotopic preference. This preference primarily originates from differences in ZPE, as shown in Table 5. Due to its higher mass, ^13^CO has lower vibrational frequencies and thus a smaller ZPE than ^12^CO. Upon adsorption, this ZPE difference results in a slightly more stable bound state for ^13^CO. Therefore, the observed enthalpy differences are mainly attributed to differences in ZPE. This aligns with the concept of chemical affinity quantum sieving, where isotope selectivity arises partly from differences in ZPE. Among the five surfaces we studied, the $\left(11\bar{2}0\right)$ surface shows the largest enthalpy and ZPE difference (1.85 meV), suggesting the strongest isotopic selectivity. In contrast, the $\left(10\bar{1}1\right)$ surface shows the smallest difference, indicating a weaker separation effect between the two isotopes. This ZPE difference, though small, is sufficient for effective separation, and similar ZPE differences on the order of magnitude have been reported in carbon isotope effects, such as the ZPE difference between ^12^CH_4_ and ^12^CH_4_ has been reported as ~29.8 cm^−1^ (~3.7 meV) [45]. Furthermore, ZPE differences between ^12^C/^13^C bonds as small as 0.01–0.05 kcal/mol (0.43–2.15 meV) have been reported in enzyme-catalyzed reactions, which still can lead to a predicted ~9‰ enrichment in pyruvate [46]. The isotope differences we obtained in CO (1.51–1.85 meV) are of the same order of magnitude as those reported in the literature. This shows that even such small ZPE differences can be effectively utilized to investigate isotope fractionation, supporting the feasibility of our approach.

**Figure 6:**
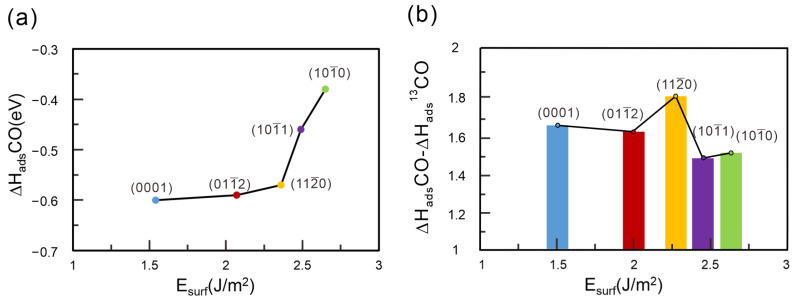
(**a**) Diagram illustrates the relationship between surface energy and adsorption enthalpy. (**b**) Bar diagram shows the relationship between surface energy and the adsorption enthalpy differences of ^12^CO and ^13^CO isotopic molecules. The blue, red, yellow, purple, and green points and bars represent the surfaces (0001), ($01\bar{1}2$), ($11\bar{2}0$), ($10\bar{1}1$), and ($10\bar{1}0$), respectively.

To better understand the surface-dependent isotope separation behavior, we systematically analyzed the adsorption characteristics of different α-Al_2_O_3_ facets, incorporating ZPE corrections, as shown in Figure 6. Figure 6a illustrates the relationship between adsorption enthalpy and surface energy. On the (0001), ($01\bar{1}2$), ($11\bar{2}0$), ($10\bar{1}1$), and ($10\bar{1}0$) surfaces, the adsorption energy difference between ^12^CO and ^13^CO primarily arises from zero-point energy differences. It shows that surfaces with lower surface energy tend to have lower adsorption enthalpy, suggesting stronger CO binding. These findings are consistent with previous studies [47,48], which reported a correlation between surface stability and adsorption strength. Figure 6b displays the differences in adsorption enthalpy between ^12^CO and ^13^CO as bars, with the corresponding ZPE differences shown as a line plot. The analysis reveals a clear trend: surfaces with lower surface energy not only adsorb CO more strongly but also exhibit larger enthalpy differences between the two CO isotopes. This suggests better isotope separation performance. Moreover, our findings support the chemical affinity quantum sieving concept, which posits that lower adsorption energy can enhance isotopic selectivity due to the greater influence of vibrational energy differences. Overall, among the various surfaces of α-Al_2_O_3_, the (0001), ($01\bar{1}2$), and ($11\bar{2}0$) planes exhibit superior separation performance. This finding provides valuable insights for future experimental design and theoretical studies, highlighting these surfaces as promising candidates for targeted investigation.

## 3. Computational Methods

All calculations were performed within the density functional theory (DFT) framework using the Vienna ab initio simulation package (VASP, version 5.4.4) [49] and the projector augmented wave (PAW) method [50]. In this work, the generalized gradient approximation (GGA) with the Perdew–Burke–Ernzerhof (PBE)-type exchange–correlation potential [51] was employed. The dispersion correction was introduced by combining Grimme’s D3 method with Becke−Johnson (BJ) damping [52]. For bulk α-Al_2_O_3_ and its 2 × 2 surface, a plane-wave cutoff of 600 eV was used. The bulk calculations adopted an 8 × 8 × 3 k-point grid, while the surface calculations used a 4 × 4 × 2 grid. The quasi-Newton algorithm as implemented in the VASP code was used in all structural relaxations. Excellent convergence of the energy differences (0.001 meV) and forces (5 meV/Å) were achieved. The optimized bulk α-Al_2_O_3_ lattice parameters were a = 4.806 Å and c = 13.113 Å, which are in excellent agreement with experimental data [53] and previously reported DFT-GGA results [54]. During the optimization of the α-Al_2_O_3_ surface model, the ionic positions, cell volume, and cell shape were allowed to relax. To account for isotopic effects, the mass parameter in the POTCAR file was modified from the default 12.011 amu (^12^C) to 13.011 amu (^13^C). This adjustment affects vibrational frequencies and zero-point energy but does not alter the electronic wavefunctions since the pseudopotential remains unchanged.

## 4. Conclusions

In this study, we constructed surface models of various low-index α-Al_2_O_3_ planes and investigated their CO adsorption and isotope separation behavior using DFT-D3(BJ) calculations with zero-point energy and van der Waals corrections. On the (0001) surface, the most favorable configuration was identified as vertical adsorption of CO via the carbon atom onto an Al site. Charge analysis and PDOS indicate weak electronic interactions with minor charge transfer, consistent with physisorption dominated by dispersion forces. NCI analysis further supports this, revealing weak, non-covalent interactions without significant repulsion or strong bonding. Across different surfaces, those with lower surface energy showed both lower adsorption enthalpy and more pronounced differences between ^12^CO and ^13^CO adsorption, suggesting better potential for isotope separation. In particular, the (0001), ($11\bar{2}0$), and ($01\bar{1}2$) surfaces exhibit the most promising separation performance. Overall, this work provides a clear understanding of how surface orientation influences CO adsorption and isotope selectivity on α-Al_2_O_3_. These insights offer useful guidance for designing materials for isotope separation and related applications.

## Figures and Tables

**Table 1:** The surface energy ($E_{surf}$, J/m^2^) of low-index α-Al_2_O_3_ surfaces.

Reference	(0001)	($11\bar{2}$0)	($01\bar{1}$2)	($10\bar{1}$1)	($10\bar{1}$0)
[30]	1.76	1.86	-	2.55	1.40
[32]	1.85	2.39	-	2.53	2.44
[33]	2.03	2.50	1.58	2.52	2.23
[35]	1.42	-	-	-	-
[36]	1.54	-	-	-	-
[37]	1.92	2.30	1.16	-	-
This work	1.54	2.36	2.07	2.49	2.65

**Table 2:** The adsorption enthalpy $∆H_{ads}$ and zero-point energy difference $∆E_{ZPE}$ of CO molecules at nine adsorption sites on an α-Al_2_O_3_ (0001) surface.

Adsorption Site	α-Al_2_O_3_/CO	α-Al_2_O_3_/^12^CO		α-Al_2_O_3_/^13^CO	
	$E_{int}(eV)$	$∆E_{ZPE}(eV)$	$∆H_{ads}(eV)$	$∆E_{ZPE}(eV)$	$∆H_{ads}(eV)$
a	−0.6595	0.0560	−0.6035	0.0543	−0.6051
b	−0.1813	0.0187	−0.1626	0.0183	−0.1630
c	0.0114	0.0172	0.0286	0.0169	0.0283
d	−0.0858	0.0155	−0.0702	0.0152	−0.0705
e	−0.6292	0.0538	−0.5754	0.0524	−0.5768
f	−0.1165	0.0357	−0.0808	0.0351	−0.0814
g	−0.4268	0.0389	−0.3878	0.0379	−0.3889
h	−0.14167	0.0275	−0.1141	0.0269	−0.1147
i	−0.1051	0.0230	−0.0821	0.0225	−0.0826

**Table 3:** Valence charge for O and C atoms in CO based on Bader charge analysis.

Adsorption Site	O(e)	C(e)	CO(e)
a	−1.05	+1.02	−0.03
b	−1.11	+1.09	−0.01
c	−1.06	+1.04	−0.02
d	−1.07	+1.04	−0.03
e	−1.07	+1.02	−0.05
f	−1.25	+1.17	−0.08
g	−1.13	+1.10	−0.03
h	−1.10	+1.05	−0.04
i	−1.14	+1.12	−0.02

**Table 4:** Adsorption enthalpy of α-Al_2_O_3_/^12^CO and α-Al_2_O_3_/^13^CO systems, including the difference in adsorption enthalpy between α-Al_2_O_3_/^12^CO and α-Al_2_O_3_/^13^CO.

Surface	$E_{int}(eV)$	$∆H_{ads}(eV)$		∆HadsCO−∆HadsCO13(meV)
	α-Al_2_O_3_/CO	α-Al_2_O_3_/^12^CO	α-Al_2_O_3_/^13^CO	
(0001)	−0.659499	−0.603479	−0.605166	1.69
$(01\bar{1}$2)	−0.631939	−0.574888	−0.576545	1.66
$(11\bar{2}$0)	−0.662079	−0.591091	−0.592936	1.85
$(10\bar{1}$1)	−0.511349	−0.459618	−0.461132	1.51
$(10\bar{1}$0)	−0.441109	−0.386177	−0.387719	1.54

**Table 5:** Zero-point energy correction of the α-Al_2_O_3_/^12^CO and α-Al_2_O_3_/^13^CO systems, including the difference in ZPE between α-Al_2_O_3_/^12^CO and α-Al_2_O_3_/^13^CO.

Surface	$∆E_{ZPE}(eV)$		∆EZPECO−CO13(meV)
	α-Al_2_O_3_/^12^CO	α-Al_2_O_3_/^13^CO	
(0001)	0.056020	0.054333	1.69
$(01\bar{1}$2)	0.057051	0.055394	1.66
$(11\bar{2}$0)	0.070988	0.069143	1.85
$(10\bar{1}$1)	0.054932	0.053390	1.51
$(10\bar{1}$0)	0.051731	0.050217	1.54

## Data Availability

The original contributions presented in this study are included in the article. Further inquiries can be directed to the corresponding authors.

## Supplementary Materials

The following supporting information can be downloaded at: https://www.mdpi.com/article/10.3390/molecules30092067/s1, Figure S1: $E_{surf}$ as a function of the number of Al-O-Al layers *n* in the slab (with *n* = 4–10) for (0001).
