# Supplementary figures and images for: Surface-Dependent Isotopic Adsorption of CO on α-Al2O3: Role of Weak Interactions and Zero-Point Energy
Source: Molecules. 2025 May 6;30(9):2067. doi: 10.3390/molecules30092067 (PMC12073176; doi:10.3390/molecules30092067)

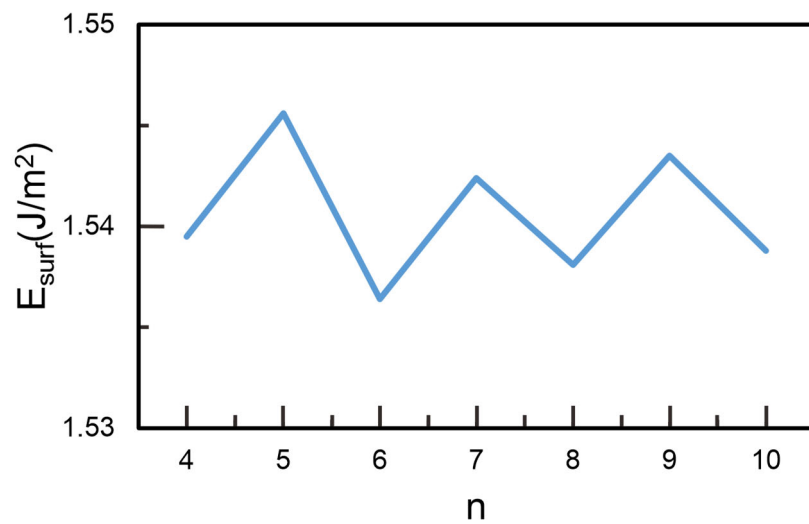

**Figure S1.**  $E_{surf}$  as a function of the number of Al-O-Al layers  $n$  in the slab (with  $n = 4-10$ ) for (0001).

Supplement: Supplementary file 1 [file molecules-30-02067-s001.zip › molecules-3624649-supplementary.pdf]
